# Th1 and Innate Lymphoid Cells Accumulate in Primary Sclerosing Cholangitis-associated Inflammatory Bowel Disease

**DOI:** 10.1093/ecco-jcc/jjx050

**Published:** 2017-04-05

**Authors:** Agnes Gwela, Priya Siddhanathi, Roger W Chapman, Simon Travis, Fiona Powrie, Carolina V Arancibia-Cárcamo, Alessandra Geremia

**Affiliations:** a Translational Gastroenterology Unit, Nuffield Department of Medicine, University of Oxford, Oxford, UK; b Kennedy Institute of Rheumatology, University of Oxford, Oxford, UK

**Keywords:** Primary sclerosing cholangitis, inflammatory bowel disease, immune response

## Abstract

**Background and Aims:**

Primary sclerosing cholangitis [PSC] is an idiopathic chronic disorder of the hepatobiliary system associated with inflammatory bowel disease [IBD], mainly ulcerative colitis [UC]. Colitis in patients with PSC and UC [PSC-UC] exhibits characteristic features and is linked to increased colon cancer risk. Genetic studies have identified immune-related susceptibility genes that only partially overlap with those involved in IBD. These observations suggest that PSC-UC may represent a distinct form of IBD. It remains to be elucidated whether different immune mechanisms are involved in colitis in these patients. We aimed to evaluate systemic and intestinal T cell and innate lymphoid cell [ILC] responses, previously associated with IBD, in patients with PSC-UC compared with patients with UC and healthy controls.

**Methods:**

Blood samples and colorectal biopsies were collected from patients with PSC-UC, patients with UC, and healthy controls. T cell and ILC phenotypes were analysed by multicolour flow cytometry.

**Results:**

Chemokine receptor [CCR] profiling of circulating T cells showed decreased CCR6^-^CXCR3^+^ Th1 cells in PSC-UC, but increased CCR6^-^CCR4^+^ Th2 cells only in UC, whereas increased CCR6^+^CCR4^+^ Th17 cells were found in both patient groups compared with healthy controls. Increased frequencies of IFN-γ secreting T cells were found in the colon of patients with PSC-UC compared with UC. Interestingly, we observed accumulation of ILC in the colon in PSC-UC.

**Conclusions:**

Our study suggests that PSC-UC represents a different immunological disorder from UC, characterised by increased intestinal Th1 and ILC responses. These results provide further evidence that PSC-UC may represent a distinct form of IBD.

## 1. Introduction

Primary sclerosing cholangitis [PSC] is a chronic progressive disorder of the hepatobiliary system characterised by inflammation, fibrosis, and stricturing of the intrahepatic and extrahepatic bile ducts, leading to liver cirrhosis. There is no cure for PSC and prognosis remains poor. PSC is associated with inflammatory bowel disease [IBD] in 60–80% of patients, among whom 80% are diagnosed with ulcerative colitis [UC] and the remaining with Crohn’s disease [CD] or indeterminate colitis.^1^ However, multiple studies have suggested that the clinical manifestations of PSC-UC differ from those of UC. In particular, PSC-UC has been reported more frequently to have a quiescent course, extensive or right-sided colitis, patchy inflammation, backwash ileitis, and higher occurrence of pouchitis after colectomy.^2,3^ Furthermore, patients with PSC-UC have a 5-fold increased risk to develop colorectal cancer compared with those with UC, and patients with PSC are also at increased risk of hepatobiliary malignancies, particularly cholangiocarcinoma [cumulative lifetime incidence 10–30%] and gallbladder adenoma, dysplasia, or carcinoma.^4–6^

As with IBD, the pathogenesis of PSC is unknown. The two main theories─which are not mutually exclusive─implicate an aberrant homing of gut-derived memory T cells to the liver, and bacterial translocation secondary to increased intestinal permeability [‘leaky gut’ hypothesis].^7,8^ Against the latter theory, however, intestinal inflammation is usually mild in these patients. More recently a PSC microbiota hypothesis has been proposed, which suggests that intestinal dysbiosis may play a central role.^9,10^

Genetic studies have identified 23 loci associated with disease susceptibility, with genome-wide significance and other suggestive associations.^11–16^ Most of these genes encode for proteins involved in immune functions and have been implicated in other immune-mediated disorders, not only IBD but also type I diabetes, coeliac disease, and rheumatoid arthritis. In particular, the strong human leukocyte antigen [HLA] association suggests that antigen-driven adaptive immune responses, possibly mediated by CD4^+^ T cells, may contribute to disease pathogenesis.^11^

The observation that only a minority of these variants are associated with IBD is surprising, considering the high concomitance of PSC and IBD, as well as the higher numbers of cases and 10 times more risk loci identified in the IBD genome-wide association studies.^17,18^ Distinct clinical manifestations and moderate genetic overlap indicate that PSC-associated IBD represents a unique form of IBD.^2^ However, the immunopathogenesis of intestinal inflammation in PSC-UC has not been fully investigated, and it remains unknown whether PSC-UC and UC are immunologically different.

We hypothesised that different lymphocyte populations may be recruited from the circulation to the colon─and potentially to the liver─to mediate inflammation and inflammation-associated cancer in patients with PSC-UC.

We analysed T cell and innate lymphoid cell [ILC] responses, previously associated with IBD, in the systemic and intestinal immune response in patients with PSC-UC compared with patients with UC and non-inflammatory controls. Increased Th1 and Th17 responses have been described in IBD patients with both CD and UC phenotypes; however, the role of increased Th1 and Th17 responses in PSC-UC has not been fully investigated.^19^ ILC are a population of lymphocytes that are enriched at mucosal sites, where they contribute to the immune response against pathogens but have also been implicated in the pathogenesis of intestinal inflammation and colitis-associated cancer in animal models.^20,21^ Several subsets of ILC reflecting functional characteristics of Th subsets have been described: T-bet^+^ ILC1 secrete IFN-γ, GATA3^+^ ILC2 secrete IL-5 and IL-13 and RORγ-t^+^ ILC3 secrete IL-17 and IL-22.^22^ Recent evidence from animal models suggests that ILC can also modulate T cell activation and proliferation through IL-2- and MHC class II-dependent interactions.^23,24^ Interestingly, we previously described an accumulation of IL-23 responsive ILC in the inflamed intestine of patients with CD, but not UC.^25^

In this study, we observed distinct chemokine receptor (CCR) profiles on circulating T cells, confirming that specific changes in T cell trafficking properties are involved in the pathogenesis of intestinal and liver inflammation in patients with PSC-UC. Furthermore, ILC accumulation and increased Th1 responses were found in the colon of these patients, where they may contribute to inflammation and increased cancer risk. Altogether our data show that PSC-UC represents an immunological disease distinct from UC.

## 2. Materials and Methods

### 2.1. Study subjects

All patients and controls were recruited from the Translational Gastroenterology Unit [TGU] in the Oxford University Hospitals and part of the Biomedical Research Centre [BRC] Oxford IBD Cohort [REC 09/H1204/30] and BRC GI Illness Biobank [REC 11/YH/0020]. Patients with a previous formal diagnosis of PSC-UC or UC, according to clinical, radiological, endoscopic and histological criteria, were identified from our databases and approached at outpatient clinic or endoscopy appointments to confirm consent and obtain blood and/or colonic samples. PSC patients with previous liver transplant were excluded from this study. Colonic biopsies were obtained from different segments of the colon [ascending, transverse, descending and rectum] from patients with PSC-UC and patients with UC alone, when undergoing colonoscopy for surveillance or disease activity and extension assessment, and from healthy controls undergoing colonoscopy for colorectal cancer screening or chronic abdominal pain/diarrhoea. Blood samples were collected from patients and healthy individuals attending the endoscopy unit, IBD clinic, or general gastroenterology clinic, as well as from healthy volunteers. Samples were used fresh in all experiments, with the exception of the intracellular TNF-α staining on peripheral blood mononuclear cells [PBMC] that was performed on previously frozen cells from a subgroup of patients and controls.

### 2.2. Cell isolation

PBMC were isolated using density centrifugation over Ficoll. Briefly, peripheral blood was diluted in an equal volume of PBS and centrifuged over a Ficoll-Hypaque layer at 2000 rpm for 20 min. Cells were collected at the Ficoll–plasma interface and washed in PBS. Lamina propria mononuclear cells [LPMC] were isolated from up to 10 biopsies using a combined mechanical and enzymatic digestion.^27^ In brief, biopsies were incubated in 1 mM DTT solution in HBSS without calcium and magnesium at room temperature on a shaker for 15 min to remove adherent mucus. After three washes, biopsies were transferred in a gentleMACS tube [Miltenyi Biotech] with 5 ml of 1 mg/ml collagenase A [Roche]/DNase solution and shaken on a gentleMACS dissociator. Samples were shaken at 37°C for 1 h and further homogenised on the gentleMACS dissociator. The digested tissue was then filtered and washed in Hanks’ balanced salt solution.

### 2.3. Cell surface and intracellular FACS characterisation

Cells were pre-incubated with 1 µg/ml of FcR blocker [Miltenyi-Biotech]. For intracellular cytokine staining, cells were first stimulated with PMA [0.1 µg/ml] and ionomycin [1 µg/ml] in the presence of Brefeldin A [3 µg/ml] for 4 h at 37°C before being surface-stained, fixed, and permeabilised [eBioscience kit]. The following antibodies were used for flow cytometry staining: intracellular staining panel including anti-CD45, anti-CD3, anti-CD8, anti-IL-17A, anti-IL-22, anti-TNF-α [all from Biolegend], and anti-IFN-γ [eBioscience] antibodies; CCR panel including anti-CXCR3 and anti-CCR4 [BD Biosciences], anti-CCR6 and anti-β7 [Biolegend], anti-CCR9 [eBioscience] and anti-CCR10 [R&D Systems] antibodies; ILC panel including anti-lineage [CD34, TCRαβ, TCRγδ, BDCA-2, FCεR1a, CD123, CD1a, CD11c, CD14, CD3, CD19, CD16], anti-CD45, anti-c-Kit, anti-CRTH2, anti-CD127 [all from Biolegend], anti-CD56 and anti-CD94 [BD Pharmingen]. All antibody cocktails contained a fluorescent dye [Invitrogen/Life technologies] to discriminate dead cells during analysis. Stained cells were acquired on an LSRII flow cytometer and analysis was performed using FlowJo software [Tree Star]. For critical discrimination, cell populations were gated against corresponding isotype or fluorescence minus one [FMO] control antibody panels.

### 2.4. Statistical analyses

Statistical analyses and graphical representations were performed using Graphpad Prism [Prism 6 software]. Data are expressed as mean ± standard error of mean [SEM]. Statistical significance between two experimental groups [PSC-UC *vs* UC, PSC-UC *vs* controls, or UC *vs* controls] was determined using the non-parametric Mann-Whitney test. *P*-values are represented on the graphs by asterisks [*] when comparing PSC-UC patients with UC patients, and by daggers [†] when comparing PSC-UC patients or UC patients with controls.

## 3. Results

### 3.1. Patients’ characteristics

The characteristics of patients and the numbers of intestinal and blood samples included in this study are shown in Table 1.

**Table 1.** Patients’ characteristics.

	**PSC-UC [*n*** = **32]**	**UC [*n*** = **34]**	**Controls [*n*** = **36]**
Blood samples, *n*	31	33	34
Colonic samples, *n*	12	9	22
Gender, males, *n* [%]	20 [62]	19 [56]	17 [47]
Age, years median [range]	52 [25–75]	51 [22–81]	62 [26–91]
Small-duct PSC, *n* [%]	4 [12]	-	-
Liver function/biochemistry tests			
ALP, median [range] IU/l	221 [117–2947]	-	-
ALT, median [range] IU/l	29.5 [13–182]	-	-
Bilirubin, median [range] µmol/l	9 [5–32]	-	-
Hyperbilirubinaemia, *n* [%] > 17 µmol/l	7 [22]		
Albumin, median [range] g/l	45 [38–49]	-	-
Platelets, median [range] x 10^9^/l	284.5 [167–607]	-	-
Thrombocytopenia, *n* [%] < 150*10^9^/l	0 [0]		
Liver cirrhosis, *n* [%]	1 [3]		
IBD duration, years median [range]	14 [3–43]	9 [0–46]	-
IBD extent			
Extensive, *n* [%]	22 [69]	9 [26]	-
Left-sided, *n* [%]	3	18 [53]	-
Proctitis, *n* [%]	0 [0]	6 [18]	-
Previous colectomy, *n* [%]	7 [22]	1 [3]	-
UCEIS			
0–2, *n*	8	7	-
3–5, *n*	4	2	-
6–8, *n*	0	0	-
Inflammation at histology			
Quiescent, *n*	8	2	-
Mild to moderate, *n*	4	7	-
Severe, *n*	0	0	-
Treatment at time of sample collection			
5-aminoslicylates, *n* [%]	20 [62]	21 [61]	-
Immunosuppressants, *n* [%]	7 [21]	10 [29]	-
Corticosteroids, *n* [%]	2 [6]	4 [11]	-
Anti-TNF-α	1 [3]	4 [11]	-
Anti-integrin therapy, *n* [%]	0 [0]	0 [0]	-
Ursodeoxycholic acid	23 [72]	-	-

PSC-UC, primary sclerosing cholangitis-ulcerative colitis; ALP, alkaline phosphatase; ALT, alanine transaminase; IBD, inflammatory bowel disease; UCEIS, Ulcerative Colitis Endoscopic Index of Severity.

There were no significant differences between UC and PSC-UC patient groups in terms of gender distribution, age, or treatment. Disease duration was similar in the two groups. The majority of PSC-UC patients presented large-duct PSC. Four blood samples and two colonic samples from four patients with small-duct [SD]-PSC-UC were also included in the analysis; 22% of PSC-UC patients had hyperbilirubinaemia. Only one patient had liver cirrhosis, as suggested by radiological tests [ultrasound or MRI] and confirmed at histological examination of liver biopsy [fibrosis staging 6/6]. As expected, a larger proportion of PSC-UC patients had extensive colitis and previous colectomy compared with UC patients. Colitis endoscopic activity was measured by the Ulcerative Colitis Endoscopic Index of Severity [UCEIS],^26^ which was comparable in the subgroups of patients with UC and PSC-UC from which biopsies were obtained. All intestinal samples presented quiescent or mild to moderate inflammation at microscopic examination. At time of sample collection, patients were receiving medical treatment as stated in Table 1.

### 3.2. Chemokine receptor expression profile on circulating T cells differs between patients with PSC-UC, patients with UC only, and controls

**Figure 1.**
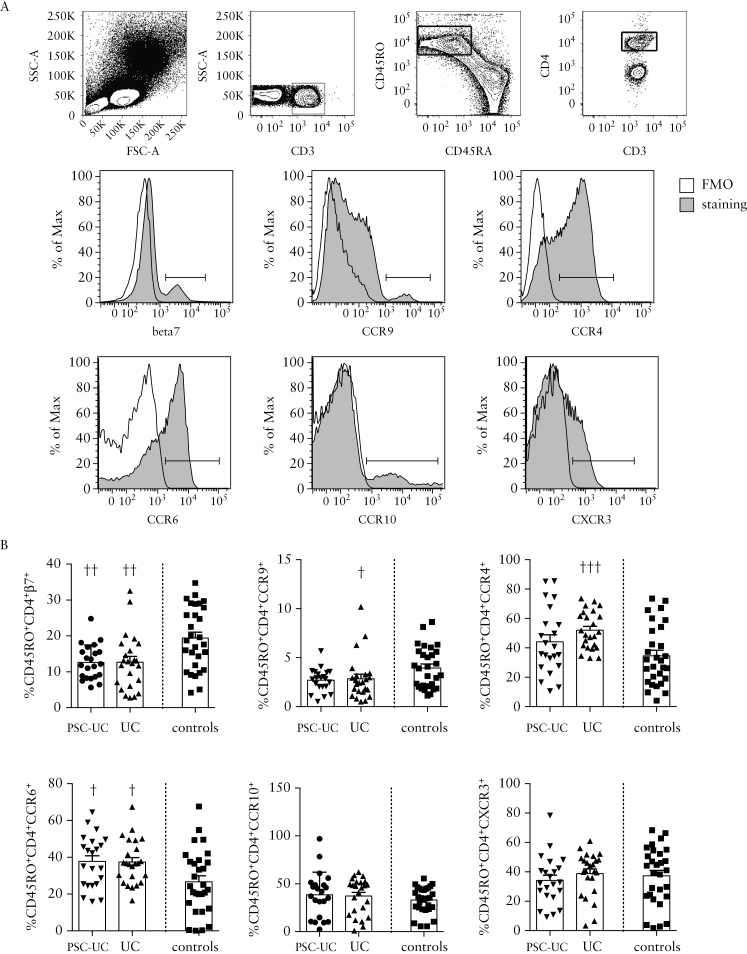
CCR expression profile of systemic T cells differs between patients with PSC-UC, UC patients, and controls. [A] Representative gating strategy. Cells were gated on lymphocytes, CD3^+^, CD45RO^+^CD45RA^-^ memory, CD4^+^ T cells. β7, CCR9, CCR4, CCR6, CCR10, and CXCR3 staining and FMO controls are shown. [B] Frequency of positive cells among CD45RO^+^CD4^+^ cells in patients with PSC-UC, UC, and controls. †*P* < 0.05, ††*P* < 0.01, †††*P* < 0.001, Mann-Whitney test *vs* controls. CCR, chemokine receptor; PSC-UC, primary sclerosing cholangitis-ulcerative colitis.

**Figure 2.**
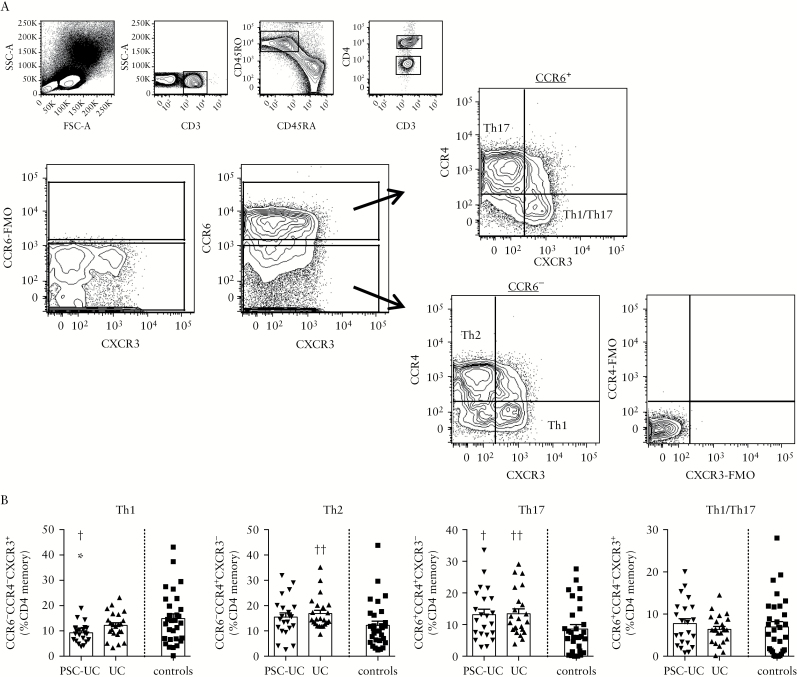
The analysis of circulating T cell subsets defined by chemokine receptor expression shows decreased Th1 in PSC-UC, increased Th2 in UC, and increased Th17 in UC and PSC-UC. [A] Representative gating strategy. Cells were gated on lymphocytes, CD3^+^, CD45RO^+^CD45RA^-^ memory, CD4^+^ T cells. CCR6^+^CXCR3^-^CCR4^+^ Th17, CCR6^+^CXCR3^+^CCR4^-^ Th1/Th17, CCR6^-^CXCR3^+^CCR4^-^ Th1, and CCR6^-^CXCR3^-^CCR4^+^ Th2 subsets were gated as shown. Gates were set using FMO controls. [B] Frequency of Th1, Th2, Th17, and Th1/Th17 as defined in [A] among the CD4 memory cells in patients with PSC-UC, UC, and controls. †*P* < 0.05, ††*P* < 0.01, Mann-Whitney test *vs* controls. PSC-UC, primary sclerosing cholangitis-ulcerative colitis; FMO, fluorescence minus one.

We evaluated T cell expression of CCR in the peripheral blood of patients with PSC-UC compared with patients with UC only and controls. The frequency of CD3^+^ T cells among lymphocytes and the proportion of CD4^+^ and CD8^+^ T cells among CD3^+^ T cells were conserved in patients with PSC-UC and patients with UC compared with healthy controls, as was expected [Supplementary Figure 1A, available as Supplementary data at *ECCO-JCC* online]. On the other hand, analysis of CCR expression on CD45RO^+^ memory CD4^+^ T cells showed a similar significant reduction in the frequency of β7^+^ cells in patients with PSC-UC and patients with UC compared with controls, supporting a central role for the α4β7 integrin in mediating the recruitment of CD4^+^ T cells to the inflamed colon in both groups of patients [Figure 1A, B]. A significant reduction in the frequency of CCR9^+^ cells was also observed in patients with UC and a similar trend was found in patients with PSC-UC, suggesting that CCR9 may also be involved in the migration of CD4^+^ T cells to the intestine and to the liver in these patients. Interestingly, the frequency of CCR4^+^ cells was exclusively increased in patients with UC compared with controls. A similar increase in the frequency of CCR6^+^ cells was found in patients with UC and PSC-UC compared with controls. No difference was observed in the frequency of CCR10^+^ and CXCR3^+^ cells between patients and controls [Figure 1A, B]. Human Th-subsets are characterised by distinct CCR expression profiles.^28^ We analysed the frequency of CCR6^-^CCR4^-^CXCR3^+^ Th1, CCR6^-^CCR4^+^CXCR3^-^ Th2, CCR6^+^CCR4^+^CXCR3^-^ Th17, and CCR6^+^CCR4^-^CXCR3^+^ Th1/Th17 among the peripheral blood memory CD4^+^ T cells in patients with PSC-UC, UC, and healthy controls. We observed decreased frequency of Th1 cells in patients with PSC-UC and increased frequency of Th2 cells in patients with UC, whereas increased frequencies of Th17 were found in both patient groups compared with controls [Figure 2A, B].

In the memory CD8^+^ T cell compartment, we observed a reduction of the β7^+^ cells in patients with UC only. Higher frequencies of CD45RO^+^CD8^+^CCR4^+^ cells were present in patients with UC compared with controls, similar to what was observed in the CD4^+^ compartment [Supplementary Figure 2, available as Supplementary data at *ECCO-JCC*].

### 3.3. Cytokines secretion by circulating T cells differs in patients with PSC-UC.

**Figure 3.**
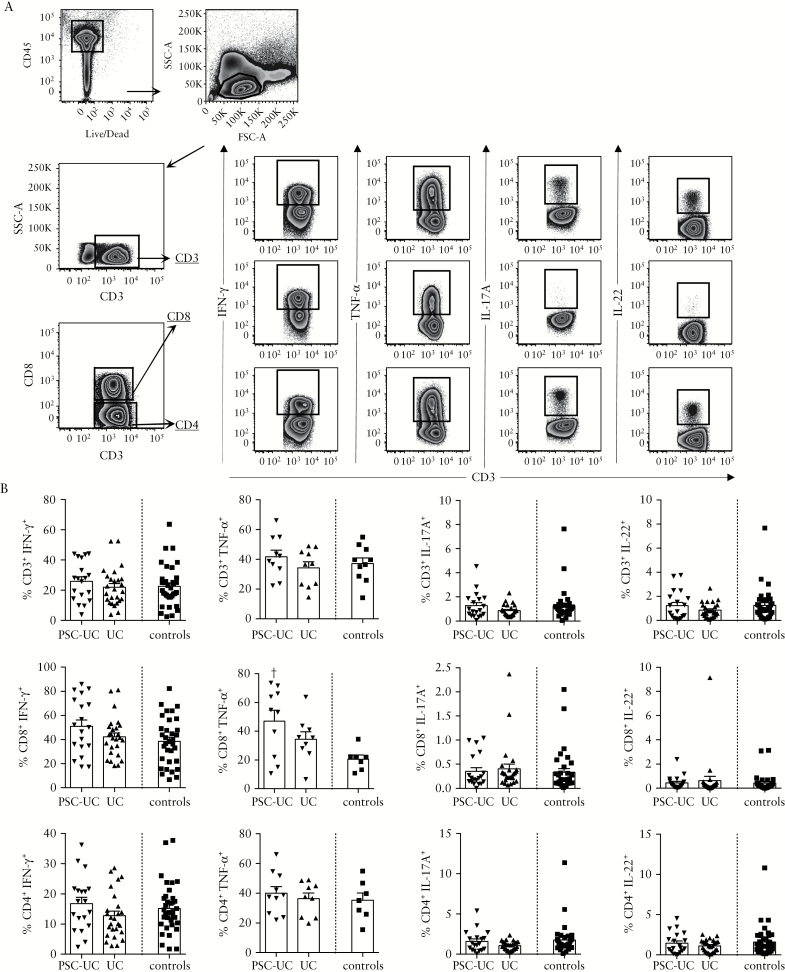
Cytokine secretion by circulating T cells in patients with PSC-UC compared with UC patients and controls. [A] Representative gating strategy for intracellular staining in PBMC. Cells were gated on CD45^+^ live cells, lymphocytes, and on CD3^+^, CD8^+^ or CD4^+^ cells. IFN-γ^+^, TNF-α^+^, IL-17A^+^, and IL-22^+^ cells were gated as shown. Gates were set using unstimulated conditions. [B] Frequency of IFN-γ^+^, TNF-α^+^, IL17-A^+^, and IL-22^+^ cells among CD3^+^, CD8^+^, and CD4^+^ cells in PBMC of patients with PSC-UC, UC and controls. †*P* < 0.05, Mann-Whitney test *vs* controls. PSC-UC, primary sclerosing cholangitis-ulcerative colitis; PBMC, peripheral blood mononuclear cells.

We then evaluated the secretion of Th1- [IFN-γ and TNF-α] and Th17- [IL-17A and IL-22] signature cytokines by peripheral blood T cells after TCR-independent stimulation. We observed increased frequency of CD8^+^TNF-α^+^ cells in PSC-UC, whereas no significant difference was observed in the frequency of IFN-γ^+^, IL-4^+^, IL-17A^+^, or IL-22^+^ cells among CD3^+^ T cells, nor in the CD8^+^ or CD4^+^ T cell subsets [Figure 3A, B].

### 3.4. The frequency of IFN-γ-secreting T cells is increased in the colon of patients with PSC-associated UC compared with patients with UC

**Figure 4.**
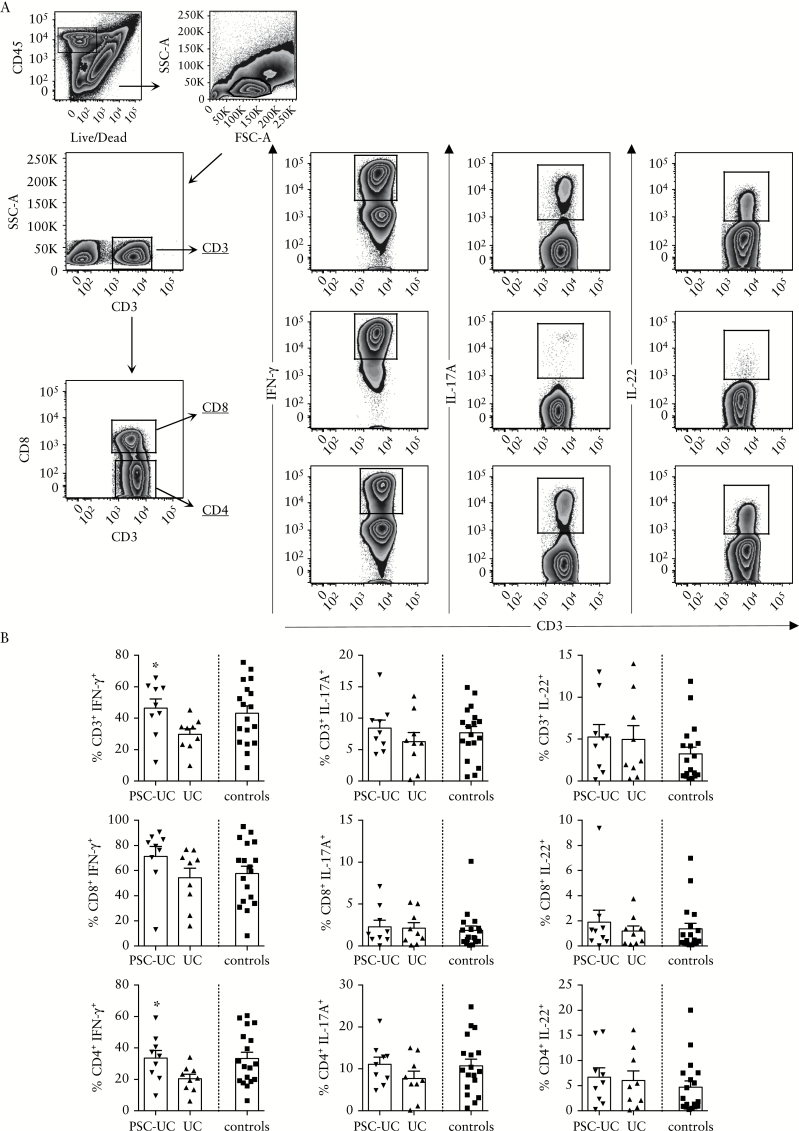
Increased frequency of IFN-γ-secreting T cells in the mucosa of patients with PSC-UC compared with UC patients. [A] Representative gating strategy for intracellular staining in LPMC. Cells were gated on CD45^+^ live cells, lymphocytes, and on CD3^+^, CD8^+^ or CD4^+^ cells. IFN-γ^+^, IL-17A^+^, and IL-22^+^ cells were gated as shown. Gates were set using unstimulated conditions. [B] Frequency of IFN-γ^+^, IL-17A^+^, and IL-22^+^ cells among CD3^+^, CD8^+^, and CD4^+^ T cells from the colon of patients with PSC-UC, UC, and controls. *P < 0.05, Mann-Whitney test *vs* UC. PSC-UC, primary sclerosing cholangitis-ulcerative colitis; LPMC, lamina propria mononuclear cells.

We evaluated the colonic T cell responses in patients with PSC-UC compared with UC patients and with controls. The frequency of intestinal CD3^+^ T cells among lymphocytes and the proportion of CD4^+^ and CD8^+^ T cells among CD3^+^ T cells were conserved in the colonic infiltrate of patients with PSC-UC and patients with UC, compared with healthy controls [Supplementary Figure 1B]. Secretion of Th1- and Th17-signature cytokines by colonic T cells was evaluated after TCR-independent stimulation. We observed a significant increase in the frequency of IFN-γ^+^ cells among CD3^+^ T cells in patients with PSC-UC compared with patients with UC only. A significant increase was found among CD4^+^ T cells, with a trend towards an increase also present in the CD8 compartment [*P* = 0.05]. No significant difference was found in the frequency of IL-17A^+^ or IL-22^+^ cells among CD3^+^ T cells, nor in the CD4^+^ or CD8^+^ T cell subsets [Figure 4A, B]. These data suggest that Th1 responses may contribute to intestinal inflammation in patients with PSC-UC.

### 3.5. Innate lymphoid cells are reduced in the peripheral blood and accumulate in the colon of patients with PSC-UC

**Figure 5.**
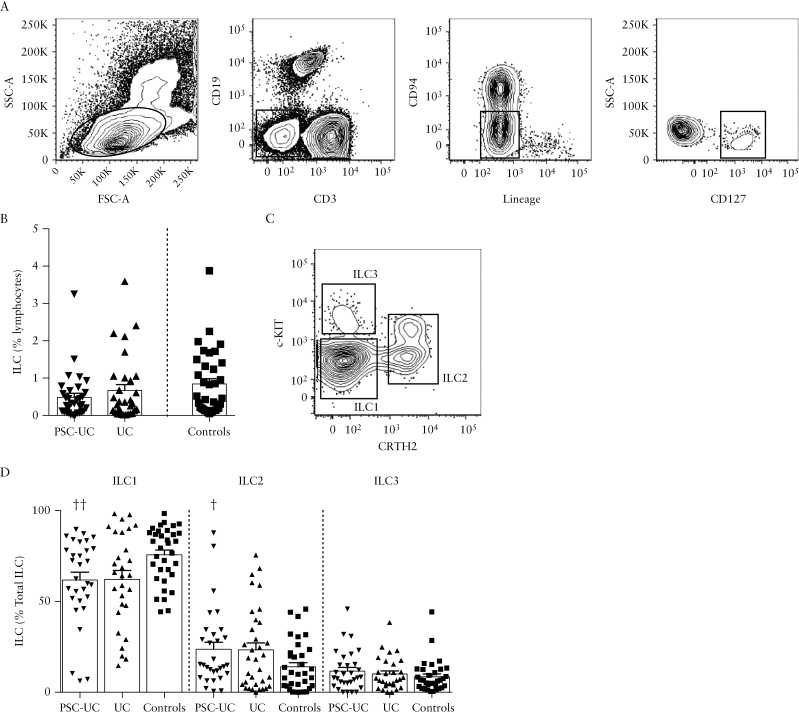
Innate lymphoid cells are reduced in the peripheral blood of patients with PSC-associated UC. [A] Representative staining of blood ILC. Cells were gated on the lymphocytic gate, CD3^-^CD19^-^, Lin^-^CD94^-^, and CD127^+^ population. [B] Frequency of ILC relative to lymphocytes in the blood of patients with PSC-UC, UC, and controls. [C] Representative staining for the ILC1, ILC2, and ILC3 subsets in the blood. After gating on ILC, as defined in [A], CRTH2^-^c-Kit^-^ ILC1, CRTH2^+^c-Kit^+/-^ ILC2, and CRTH2^-^c-Kit^+^ ILC3 were gated as shown. [D] Frequency of ILC1, ILC2, and ILC3 among total ILC in the blood of patients with PSC-UC, UC, and controls. †*P* < 0.05, ††*P* < 0.01, Mann-Whitney test *vs* controls. PSC-UC, primary sclerosing cholangitis-ulcerative colitis; ILC, innate lymphoid cell.

**Figure 6.**
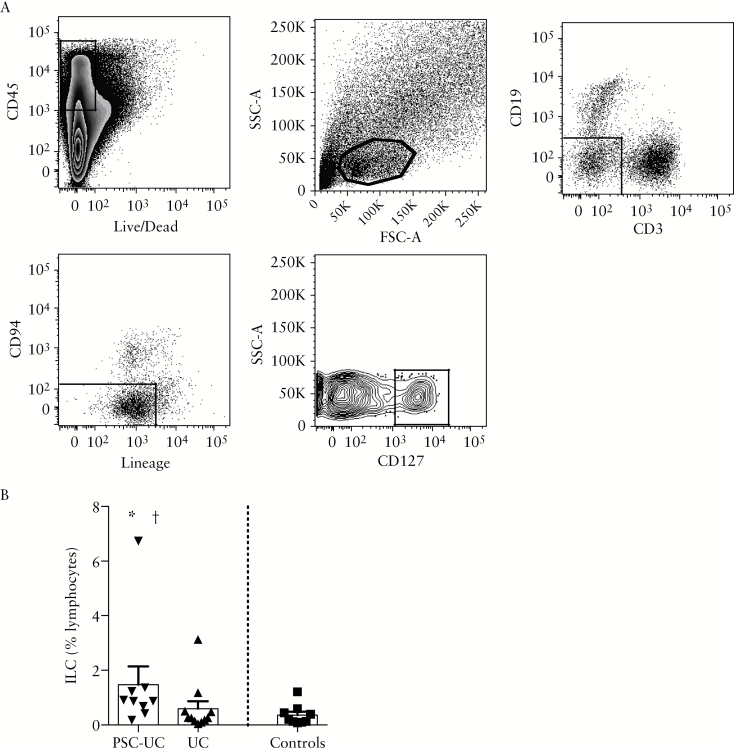
Innate lymphoid cells accumulate in the colon of patients with PSC-UC. [A] Representative staining of colonic ILC. Cells were gated on the CD45^+^ alive cells, the lymphocytic gate, CD3^-^CD19^-^, Lin^-^CD94^-^, and CD127^+^ population. [B] Frequency of ILC relative to lymphocytes in the colon of patients with PSC-UC, UC, and controls. *P < 0.05, Mann-Whitney test *vs* UC; †*P* < 0.05, Mann-Whitney test *vs* controls. PSC-UC, primary sclerosing cholangitis-ulcerative colitis; ILC, innate lymphoid cell

We analysed the frequency of ILC among lymphocytes in peripheral blood and observed a trend towards a reduction in the frequency of ineage^-^ CD127^+^ ILC in peripheral blood of patients with PSC-UC compared with controls [Figure 5A, B, *P* = 0.05]. We characterised the frequency of c-KIT^-^CRTH2^-^ ILC1, CRTH2^+^ ILC2 and c-KIT^+^CRTH2^-^ ILC3 cells among total ILC, and found a reduced proportion of ILC1 and increased frequency of ILC2 in the peripheral blood of patients with PSC-UC compared with controls, with a similar trend in patients with UC [Figure 5C, D]. Interestingly, we observed an increased frequency of lineage^-^CD127^+^ ILC in the colon of patients with PSC-UC compared with patients with UC and with controls [Figure 6A, B]. These data suggest that ILC may contribute to colonic inflammation and increased risk of cancer in PSC-UC.

## 4. Discussion

It has long been debated whether PSC-associated IBD should be considered a distinct form of IBD. Colitis in PSC has classically been related to that in UC, even if it has become clear that the clinical phenotype of colitis in PSC is unique. It is usually characterised by an indolent course of pancolitis or right-sided disease, with more frequent rectal sparing and backwash ileitis, higher incidence of pouchitis after colectomy, and a 5-fold increased risk of colorectal cancer compared with patients with UC.

Results of genetic studies have shown only partial overlap in the genetic susceptibility risks between PSC and IBD, supporting the hypothesis that these two diseases may represent distinct immunological disorders. More recently, the analysis of the intestinal microbiota has also identified specific gut microbial signatures in PSC compared with patients with UC and with healthy controls, again distinguishing patients with PSC [with or without IBD] from patients with UC.^9,10^ Altogether, this line of evidence indicates that intestinal inflammation in PSC may be pathogenetically different from that in UC. However, only a very limited number of studies have attempted to analyse the intestinal immune response in PSC-associated IBD.^29,30^ Therefore, it remains to be elucidated whether the immunological mechanisms involved in intestinal inflammation in PSC-associated colitis differ from those involved in IBD and more specifically UC. In this study, we investigated the systemic and intestinal immune response in patients with PSC-UC compared with patients with UC only and with healthy controls.

Macroscopic and microscopic intestinal inflammation was comparable in those patients from whom biopsies were obtained and the mucosal immune response was analysed.

The CCR profiling of peripheral memory T cells confirmed a role for the α4β7 integrin and CCR9 in the migration of T cells to the inflamed colon–and possibly to the liver–in patients with PSC-UC. In fact, previous studies have shown that the respective ligands, MAdCAM-1 and CCL25, normally expressed on the intestinal mucosal vessels, are ectopically expressed in the liver in PSC, and 20% of liver-infiltrating lymphocytes express CCR9 and α4β7.^7^ We observed an increased frequency of CCR4^+^ CD4^+^ and CD8^+^ T cells in UC, but not in PSC-UC, compared with healthy controls. On the other hand, CCR6^+^ CD4^+^ T cells were increased in both patient groups compared with controls.

Differences in CCR expression may reflect Th polarisation between PSC-UC and UC immune responses. Activated CD4^+^ T cells, after exposure to different inflammatory signals, express lineage-specific transcription factors and differentiate in Th subsets characterised by distinct trafficking capacities and immune effector functions.^28^ Classical Th1 cells are CXCR3^+^, express T-bet, and secrete IFN-γ. Th1 cells participate in the immune response towards intracellular pathogens and have been implicated in the pathogenesis of multiple immune disorders including IBD. We observed decreased frequencies of CCR6^-^CCR4^-^CXCR3^+^ Th1-enriched cells in PSC-UC, but no differences were found in Th1 cytokine [IFN-γ and TNF-α] secretion. CCR4 [in the absence of CCR6] is predominantly expressed by Th2 cells, which secrete IL-4, IL-5, and IL-13. Th2 responses mediate protection from parasites, but are also implicated in allergic reactions. Overactive atypical Th2 responses were originally described in UC, but subsequent studies have challenged the notion that UC is a Th2-driven disease.^19^ Our Th-subset analysis based on CCR profiling showed increased frequency of CCR6^-^CCR4^+^CXCR3^-^ Th2 cells in UC. CCR4 together with CCR6 is also expressed on Th17 cells, which are RORγ^+^, secrete IL-17 and IL-22, and participate in the response to extracellular bacteria and fungi. Furthermore, a population of CCR6^+^CCR4^-^CXCR3^+^ Th1/Th17 cells has been described which express both T-bet and RORγ, produce IL-17 and IFN-γ, and may play a pathogenic role in IBD.^31–35^ Increased Th17 responses are found in IBD and have also been associated with liver inflammation and PSC.^19,36^ The frequency of circulating CCR6^+^CCR4^+^CXCR3^-^ Th17 cells was higher in both patients with PSC-UC and those with UC only compared with controls, whereas no differences were observed in the Th1/Th17 subset. No difference was found in IL-17 or IL-22 secretion by systemic T cells after stimulation.

The discrepancy observed between systemic T cell subsets frequencies and cytokine secretion by circulating T cells may reflect the stimulation with membrane-permeable activators of the protein kinase C, whereas more physiological TCR-stimulation may be required to detect functional differences in the periphery. However, when colonic T cells were stimulated with PMA/ionomoycin, increased frequencies of IFN-γ secreting T cells were found in patients with PSC-UC compared with patients with UC. This finding, together with the reduced frequency of CCR6^-^CCR4^-^CXCR3^+^ Th1 cells in the peripheral blood in PSC-UC, may indicate that Th1 cells are recruited to the colon in PSC-UC where they can contribute to intestinal inflammation through the production of IFN-γ and other pro-inflammatory cytokines.

We then evaluated whether ILC populations, previously associated with IBD pathogenesis, are differentially represented in the systemic and intestinal immune responses in patients with PSC-UC and UC. ILC have been shown to play a central role in driving intestinal inflammation in animal models of colitis, and we have previously shown that they accumulate in the inflamed intestine of patients with CD, but not those with UC.^20, 25^ Interestingly, IL-22-secreting ILC can also drive colitis-associated cancer through the production of IL-22 in murine models.^21^

In this study, we observed a trend towards a reduced frequency of ILC in the peripheral blood and significant accumulation of ILC in the colon of patients with PSC-UC, but not UC only, compared with controls. The ILC subset analysis in the peripheral blood may indicate that ILC1 are specifically recruited and contribute to tissue inflammation. However, a degree of plasticity has been described between ILC1 and ILC3 populations, depending on the inflammatory milieu.^37–39^ ILC3 can differentiate towards ILC1 in presence of IL-12, while IL-23, IL-1β, and IL-2 can induce differentiation of ILC1 into ILC3 *in vitro*, with the vitamin A metabolite retinoic acid also contributing to the differentiation of ILC1 towards ILC3. The accumulation of ILC in the colon of patients with PSC-UC may contribute to tissue inflammation and increased cancer risk in these patients through cytokine production and the interaction with immune and non-immune cells. However, we appreciate that the low number of colonic samples represents a limitation of this study. Therefore, further work will be needed to confirm these observations.

PSC-CD patients were not included in this study in view of previous analysis of the Oxford PSC patients’ cohort, which showed a distinct clinical phenotype of patients with PSC-CD compared with PSC-UC. In particular Halliday *et al.* have shown that PSC-CD patients were as likely to be female as male, more commonly had small-duct PSC and less commonly progressed to cancer, liver transplantation, or death.^40^ These observations were recently confirmed in a large retrospective outcome analysis.^41^ Recent results of genetic studies identify PSC-IBD as a single entity, and future work should address whether there are any differences in the immune response associated with colitis in PSC-CD *vs* PSC-UC.

In this study we have identified different T cell- and ILC-mediated immune responses in the peripheral blood and colon of patients with PSC-UC, supporting our hypothesis that PSC-associated colitis represents a distinct immunological disorder from UC. These findings are in agreement with the results of the genetic studies that have shown low genetic correlations between PSC and UC or CD and have identified a strong HLA class II association in PSC, supporting a role for T cell responses in the pathogenesis of this disease.

The identification of specific immune pathways involved in PSC-associated IBD may improve patient stratification and early diagnosis, and provide potential therapeutic targets for treatment.

## Funding

This work was supported by Crohn’s and Colitis UK [grant number M/13/3]; and the Wellcome Trust [grant number 101734/Z/13/Z].

## Conflict of Interest

The authors have no conflicting financial interests.

## Author Contributions

AG: technical and material support, acquisition of data, analysis and interpretation of data, statistical analysis, drafting of the manuscript. PS: technical and material support. RWC: material support, critical revision of the manuscript. ST: material support, critical revision of the manuscript. FP: material support, critical revision of the manuscript. CVA: material support, interpretation of data, critical revision of the manuscript. AG: study concept and design, acquisition of data, analysis and interpretation of data, statistical analysis, drafting and critical revision of the manuscript.

## Supplementary Data

Supplementary data are available at *ECCO-JCC* online.

## Supplementary Material

Supplementary Figures 1 and 2
