# Supplementary material for: Th1 and Innate Lymphoid Cells Accumulate in Primary Sclerosing Cholangitis-associated Inflammatory Bowel Disease
Source: J Crohns Colitis. 2017 Apr 5;11(9):1124–34. doi: 10.1093/ecco-jcc/jjx050 (PMC5637950; doi:10.1093/ecco-jcc/jjx050)
Supplement: Supplementary Figures 1 and 2 [file jjx050_suppl_supplementary_figures_1-2.docx]

**Supplementary Figure Legends**

**Supplementary Figure 1. Maintained composition of T-cell infiltrate in PSC-UC compared to UC and controls.** (A) Frequency of CD3^+^, amongst total lymphocyte, CD4^+^ and CD8^+^ cells relative to CD3^+^ cells in the blood of patients with PSC-UC, UC patients and controls. (B) Frequency of CD3^+^ relative to total lymphocytes and CD4^+^ and CD8^+^ cells amongst CD3^+^ cells in the colon of patients with PSC-UC, UC patients and controls.

**Supplementary Figure 2.** **Chemokine receptor expression profile of CD8^+^ T-cells differs between patients with PSC-UC, UC patients and controls.** Frequency of positive cells amongst CD45RO^+^CD8^+^ cells in the blood of patients with PSC-UC, UC and controls. † p<0.05, †† p<0.01 , Mann-Whitney test *vs.* controls.

**Supplementary Figure 1**

A

B

**Supplementary Figure 2**
